# Genomic Features of Response to Combination Immunotherapy in Patients with Advanced Non-Small-Cell Lung Cancer

**DOI:** 10.1016/j.ccell.2018.03.018

**Published:** 2018-05-14

**Authors:** Matthew D. Hellmann, Tavi Nathanson, Hira Rizvi, Benjamin C. Creelan, Francisco Sanchez-Vega, Arun Ahuja, Ai Ni, Jacki B. Novik, Levi M.B. Mangarin, Mohsen Abu-Akeel, Cailian Liu, Jennifer L. Sauter, Natasha Rekhtman, Eliza Chang, Margaret K. Callahan, Jamie E. Chaft, Martin H. Voss, Megan Tenet, Xue-Mei Li, Kelly Covello, Andrea Renninger, Patrik Vitazka, William J. Geese, Hossein Borghaei, Charles M. Rudin, Scott J. Antonia, Charles Swanton, Jeff Hammerbacher, Taha Merghoub, Nicholas McGranahan, Alexandra Snyder, Jedd D. Wolchok

**Affiliations:** 1 Department of Medicine, Memorial Sloan Kettering Cancer Center, 885 2^nd^ Avenue, New York, NY 10017, USA; 2 Weill Cornell School of Medicine, New York, NY, USA; 3 Druckenmiller Center for Lung Cancer Research, Memorial Sloan Kettering Cancer Center, New York, NY, USA; 4 Parker Institute for Cancer Immunotherapy, Memorial Sloan Kettering Cancer Center, New York, NY, USA; 5 Department of Genetics and Genomic Sciences, Icahn School of Medicine at Mount Sinai, New York, NY, USA; 6 Department of Immunology, H. Lee Moffitt Cancer Center and Research Institute, Tampa, FL, USA; 7 Human Oncology and Pathogenesis Program, Memorial Sloan Kettering Cancer Center, New York, NY, USA; 8 Marie-Josèe and Henry R. Kravis Center for Molecular Oncology, Memorial Sloan Kettering Cancer Center, New York, NY, USA; 9 Department of Biostatistics and Epidemiology, Memorial Sloan Kettering Cancer Center, New York, NY, USA; 10 Ludwig Collaborative Laboratory, Memorial Sloan Kettering Cancer Center, New York, NY, USA; 11 Department of Pathology, Memorial Sloan Kettering Cancer Center, New York, NY, USA; 12 Bristol Myers Squibb, Princeton, NJ, USA; 13 Fox Chase Cancer Center, Philadelphia, PA, USA; 14 Cancer Research UK Lung Cancer Centre of Excellence, University College London Cancer Institute, London, UK; 15 Translational Cancer Therapeutics Laboratory, Francis Crick Institute, London, UK; 16 Department of Microbiology and Immunology, Medical University of South Carolina, Charleston, SC, USA

**Keywords:** immunotherapy, mutation burden, TMB, lung cancer, PD-1, CTLA-4

## Abstract

Combination immune checkpoint blockade has demonstrated promising benefit in lung cancer, but predictors of response to combination therapy are unknown. Using whole-exome sequencing to examine non-small-cell lung cancer (NSCLC) treated with PD-1 plus CTLA-4 blockade, we found that high tumor mutation burden (TMB) predicted improved objective response, durable benefit, and progression-free survival. TMB was independent of PD-L1 expression and the strongest feature associated with efficacy in multivariable analysis. The low response rate in TMB low NSCLCs demonstrates that combination immunotherapy does not overcome the negative predictive impact of low TMB. This study demonstrates the association between TMB and benefit to combination immunotherapy in NSCLC. TMB should be incorporated in future trials examining PD-(L)1 with CTLA-4 blockade in NSCLC.

## Highlights

• High TMB correlates with efficacy of PD-1 plus CTLA-4 blockade in NSCLC
• TMB and PD-L1 are independent variables
• In multivariate analysis, TMB associated most strongly with efficacy

## Teaser

Hellmann et al. examine non-small-cell lung cancers treated with combined PD-1 and CTLA-4 blockade using whole-exome sequencing and find that high tumor mutation burden is the strongest feature associated with improved objective response, durable benefit, and progression-free survival in multivariable analysis.

## Significance

**Our study examines the molecular features associated with response in patients with NSCLC treated with the combination of PD-1 plus CTLA-4 blockade. Contrary to our initial hypothesis, tumor mutation burden is the strongest feature associated with benefit. Combination immunotherapy may be particularly effective in those with high TMB but is insufficient to overcome the negative predictive impact of low mutation burden. This report highlights the critical importance of tumor mutation burden as a predictive marker and provides insight into the determinants of response in those treated with combination immune checkpoint blockade therapy.**

## Introduction

T cell checkpoint inhibitors have improved the survival of patients with a multitude of advanced malignancies. Antibodies targeting programmed cell death receptor-1 (PD-1) or its ligand (PD-L1) are now approved for treating multiple cancers, including non-small-cell lung cancer (NSCLC) (Borghaei et al., 2015, Reck et al., 2016). Responses to anti-PD-(L)1 monotherapies have the potential for remarkable durability, but occur in only a minority of patients. This experience highlights two primary opportunities for continued progress: identification of predictive biomarkers of response and development of combinatorial treatment approaches that improve the frequency, depth, and duration of response.

Toward identification of predictors of response to anti-PD-(L)1 monotherapy, tumor expression of PD-L1 has been a primary focus. However, the sensitivity and specificity of PD-L1 expression is modest (Reck et al., 2016, Taube et al., 2014), which has prompted the search for additional predictive tools. Our group and others have identified an association between increased nonsynonymous tumor mutation burden (TMB) and response in patients with melanoma treated with CTLA-4 blockade (Snyder et al., 2014, Van Allen et al., 2015), NSCLCs treated with PD-1 blockade (Carbone et al., 2017, Rizvi et al., 2015, Rizvi et al., 2018), mismatch repair deficient tumors treated with PD-1 blockade (Le et al., 2017), and bladder cancers treated with PD-L1 blockade (Rosenberg et al., 2016).

In parallel to these correlative efforts, multiple ongoing clinical trials are attempting to improve response rates by combining immunotherapies. Pre-clinical (Wei et al., 2017) and clinical (Hammers et al., 2017, Hellmann et al., 2017, Wolchok et al., 2017) studies have identified non-redundant effects of CTLA-4 and PD-1 signaling and synergistic anti-tumor responses (Curran et al., 2010). To date, the genomic determinants of response to combination immunotherapy have not been defined.

We sought to examine the molecular features correlated with response in patients with NSCLC treated with combination immunotherapy. In particular, we focused on whether TMB would correlate with response, as it has in patients treated with PD-1 monotherapy, or if combination therapy may broaden the repertoire of effective anti-tumor immunity and thereby diminish the importance of TMB.

## Results

### Genomic and Clinical Characteristics of Study Cohort Are Generalizable

We performed whole-exome sequencing (WES) on tumor tissue and paired blood (Van Allen et al., 2014) collected from 75 patients with NSCLC treated with nivolumab plus ipilimumab as part of the CheckMate-012 study (Hellmann et al., 2017) (Table 1). The clinical features and efficacy outcomes in the cohort of patients examined with WES were similar to the overall set of patients enrolled in CheckMate-012 (Figure S1; Tables S1 and S2). The mean target coverage was 148X (interquartile range [IQR] 116-182X) in tumors and 81X (IQR 70-98X) in normal; 94% of target sequences were sequenced to at least 20X depth in tumors (Table S3). Sequencing coverage was similar between responders and non-responders (Figure S2A). TMB was defined as total number of nonsynonymous single nucleotide and indel variants (Table S3). Except for expected differences by smoking status, baseline clinical variables were similar between those with TMB above versus below median (Table 1).

**Table 1:** Baseline Clinical Characteristics

Patient Characteristics (n = 75)	All Patients	TMB Low	TMB High	p Value
	No. (%)	No. (%)	No. (%)	
Age (years), median (range)	66 (42–87)	66 (43–85)	65 (42–87)	0.7739
Gender				
Male	37 (49)	17 (45)	20 (54)	0.4916
Female	38 (51)	21 (55)	17 (46)	
Histology				
Non-squamous	59 (79)	31 (82)	28 (76)	0.5829
Squamous	16 (21)	7 (18)	9 (24)	
Smoking status				
Current/former	60 (80)	24 (63)	36 (97)	**0.0003**
Never	15 (20)	14 (37)	1 (3)	
Stage				
IIIB	9 (12)	6 (16)	3 (8)	0.4799
IV	66 (88)	32 (84)	34 (92)	
Performance status				
ECOG 0	30 (40)	16 (42)	14 (38)	0.8147
ECOG 1	45 (60)	22 (58)	23 (62)	
PD-L1 expression				
0%	25 (33)	13 (34)	12 (32)	0.8037^a^
≥1%	45 (60)	21 (55)	24 (65)	
Unknown	5 (7)	4 (11)	1 (3)	
Best overall response				
Complete/partial response	24 (32)	5 (13)	19 (51)	**0.0018**
Stable disease	27 (36)	17 (45)	10 (27)	
Progression/not evaluable	24 (32)	16 (42)	8 (22)	
Clinical benefit				
Durable clinical benefit (DCB)	37 (49)	13 (34)	24 (65)	**0.0111**
No durable benefit (NDB)	38 (51)	25 (66)	13 (35)	

p values in bold type represent significance <0.05. See also Figure S1 and Tables S1 and S2.

a Reflects comparison of PD-L1 0% versus ≥1%.

The median and distribution of TMB and transition/transversion ratio in this study were similar to NSCLC tumors sequenced as part of The Cancer Genome Atlas (TCGA) (Campbell et al., 2016) (Figure S2B and Table S3).

### Tumor Mutation Burden Is Significantly Associated with Improved Efficacy of Combination Immunotherapy

**Figure 1:**
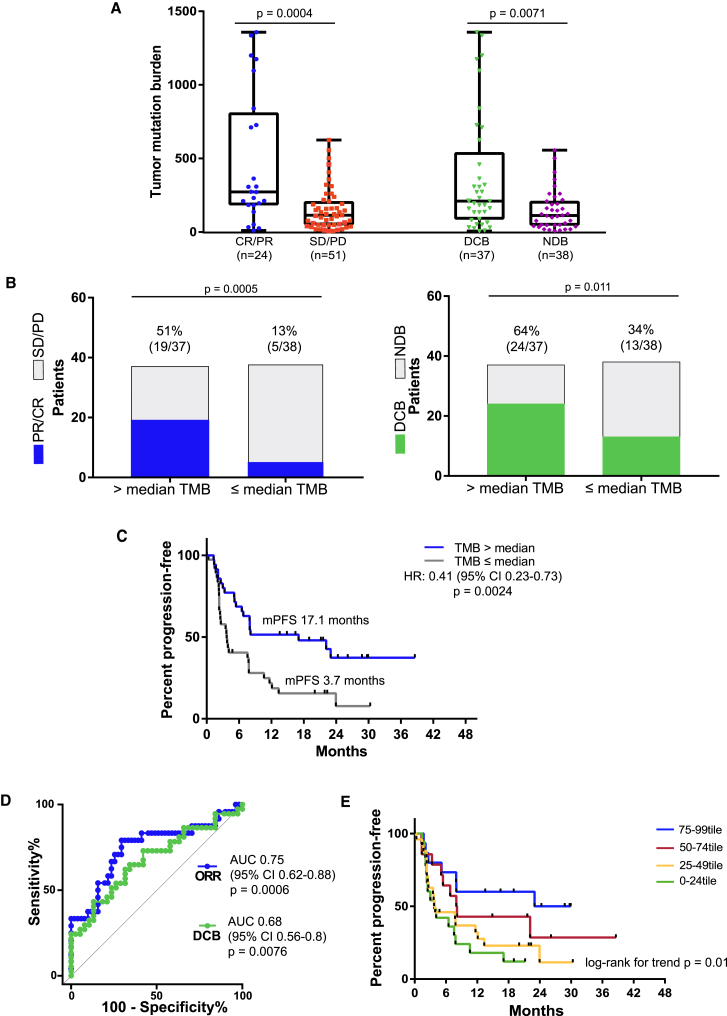
TMB Correlates with Efficacy in Patients with NSCLC Treated with Nivolumab Plus Ipilimumab (A) TMB in patients with complete response (CR)/partial response (PR) (n = 24, blue) versus stable disease (SD)/progressive disease (PD) (n = 51, red) (median 273 versus 114 mutations, Mann-Whitney p = 0.0004) and TMB in patients with DCB (green, n = 37) versus those with NDB (purple, n = 38) (median 210 versus 113 mutations, Mann-Whitney p = 0.0071). Medians, interquartile ranges, and minimum/maximum shown in boxplots. (B) Objective response and durable clinical benefit in patients with high TMB (>median, 158 mutations) versus low TMB (≤median) (ORR 51% versus 13%, odds ratio 6.97 [95% confidence interval (CI) 2.19–19.0], Fisher's exact p = 0.0005; DCB 65% versus 34%, odds ratio 3.55 [95% CI 1.3–8.64], Fisher's exact p = 0.011). Proportion of CR/PR or DCB, respectively, are colored on histograms with rate (n/N) shown above each bar. (C) PFS in patients with high TMB versus low TMB (median 17.1 versus 3.7 months, Mantel-Haenszel hazard ratio 0.41 [95% CI 0.23–0.73], log rank p = 0.0024). (D) Receiver operating characteristic (ROC) curves for correlation of TMB with objective response (CR/PR; blue line) (AUC 0.75 [95% CI 0.62–0.88], p = 0.0006) and DCB (green line) (AUC 0.68 [95% CI 0.56–0.8], p = 0.0076). (E) PFS in cohorts of patients defined by quartiles of TMB percentile rank among NSCLC tumors profiled by TCGA (log rank for trend p = 0.01). See also Figure S2 and Table S3.

TMB was higher in patients with objective response (complete or partial response) compared with those with no response (stable or progressive disease) (median TMB 273 versus 114 mutations, Mann-Whitney p = 0.0004, Figure 1A). Similar results were seen when comparing patients with durable clinical benefit (DCB; partial or stable response for >6 months) with those with no durable benefit (NDB) (median TMB 210 versus 113, Mann-Whitney p = 0.0071). Objective response rate, DCB rate, and progression-free survival (PFS) were all greater in patients with high TMB (>median, 158 mutations) compared with low TMB (≤median) (overall response rate [ORR] 51% versus 13%, Fisher's exact p = 0.0005; DCB 65% versus 34%, Fisher's exact p = 0.011; PFS hazard ratio = 0.41, log rank p = 0.0024) (Figures 1B and 1C). There was a strong association between increased TMB and increased rate of ORR or DCB (ORR area under the curve [AUC] 0.75, p = 0.0006, DCB AUC 0.68, p = 0.0076) (Figure 1D).

To contextualize this dataset and facilitate generalizability, we also characterized TMB in this cohort as a percentile rank of NSCLCs profiled by TCGA. Serial increases in percentile threshold were associated with improved PFS (Figure 1E). Patients with tumors in either the upper half or upper tertile of TMB had significantly improved ORR and PFS (Figures S2C and S2D).

### Computationally Predicted Neoantigen Burden and Mutation Burden Are Closely Correlated

Consistent with previous reports (Rizvi et al., 2015) and potentially indicative of the mechanistic importance of neoantigens generated from somatic nonsynonymous mutations (Schumacher and Schreiber, 2015), TMB (defined as nonsynonymous variants) was more strongly associated with ORR and PFS than mutation burden inclusive of silent variants (Figure S2E). However, computationally predicted candidate neoantigen burden (Nathanson et al., 2017) (Table S3) was not more predictive than TMB of clinical benefit when using either a moderate or strong threshold (500 nM or 50 nM) of neoantigen binding affinity to patient-specific class I HLA alleles (Figure S2F). Clonal predicted neoantigen burden was more predictive of improved PFS compared with total predicted neoantigen burden (> versus ≤ median predicted clonal neoantigens, log rank p = 0.04; > versus ≤ median total predicted neoantigens versus p = 0.07). TMB and computationally predicted neoantigen burden were highly correlated (Spearman ρ 0.92, p < 0.0001; Figure S2G), consistent with this characteristically proportional relationship (Rooney et al., 2015, Van Allen et al., 2015). There were no clear associations between specific HLA alleles and objective response (Figure S2H).

### Individual Genes and Additional Molecular Features Associated with Response or Resistance to Combination Immunotherapy

**Figure 2:**
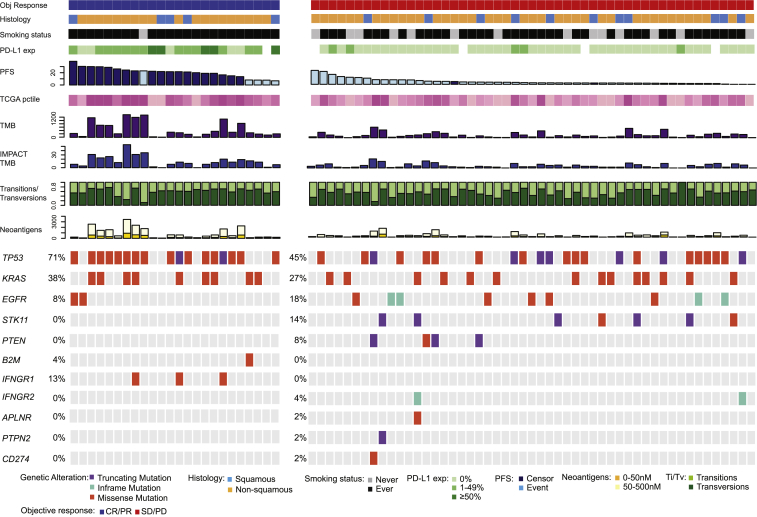
Summary of Clinical and Molecular Features Associated with Response or Non-response in Patients with NSCLC Treated with Nivolumab Plus Ipilimumab Individual patients are represented in each column, organized by those with objective response on the left (blue) and those with no objective response on the right (red). Categories of histology (squamous or non-squamous) and smoking status (never or ever) are characterized. PD-L1 expression is stratified as 0%, 1%–49%, or ≥50%. PFS is shown in months, with the color of each bar representing those who are censored (dark blue) or have progressed (light blue). The NSCLC TCGA percentile rank for each case is described from 0% to 100% in light to dark purple. Nonsynonymous TMB and mutation burden quantified using genes including in the MSK-IMPACT targeted next-generation sequencing panel are shown in histograms. The percent of transitions (light green) and transversions (dark green) are shown. Candidate neoantigen burden is quantified in histograms, stratified by predicted patient-specific HLA binding affinity 0–50 nM (orange) or 50–500 nM (light yellow). The occurrences of selected genes in each case are represented in the oncoprint, with the percent frequency in responders or non-responders shown. See also Figures S2 and S3; Tables S3 and S4.

We next explored other molecular features that may refine the association of TMB with response to combination immunotherapy (Figure 2). Pre-clinical (Burr et al., 2017, Manguso et al., 2017, Mezzadra et al., 2017, Patel et al., 2017, Skoulidis et al., 2015) and clinical reports (Gao et al., 2016, George et al., 2017, Zaretsky et al., 2016) have described associations between individual altered genes and response or resistance to immune checkpoint blockade (Table S4). Relatively few of these genes were found in this dataset (Figure 2), but some genes were exclusively associated with resistance in our series, such as *STK11* (zero responses in seven patients with *STK11* mutations) and *PTEN* (0 of 4), consistent with previous reports, although not reaching statistical significance likely owing to small numbers (Table S4). *IFNGR1* mutations (n = 3) were found only in responders. To identify other potential genes of interest, we identified significantly recurrent genes using MutSigCV (Lawrence et al., 2013) (Table S4). Of these genes, only *TP53* mutations were enriched in responders (odds ratio 2.9, Fisher's exact p = 0.048, Figures S3A and S3B). Notably, *TP53* mutations were also associated with increased mutation burden in both the cohort of combination immunotherapy NSCLCs and TCGA NSCLCs (Figures S3C–S3F and Table S4).

Additionally, to explore the applicability of targeted next-generation sequencing as an estimate of exonic mutation burden (Chalmers et al., 2017, Zehir et al., 2017), we found that limiting variants to the 468 genes represented in our institutional MSK-IMPACT panel (Zehir et al., 2017) or the 315 genes in the FoundationOne panel (Frampton et al., 2013) maintained similar predictive fidelity for efficacy (Figures S3G–S3H).

### Tumor Mutation Burden Is Independent of PD-L1 and Remains Significantly Associated with Efficacy in Multivariable Analysis

**Figure 3:**
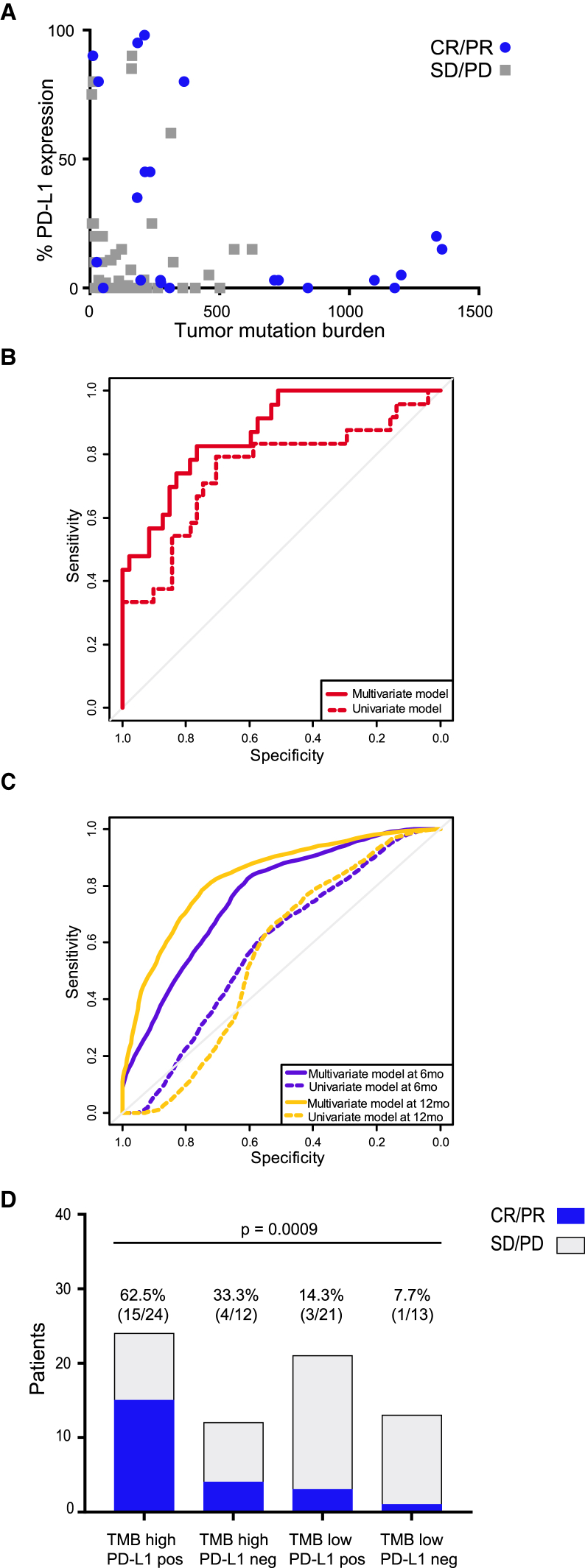
Association between TMB and Efficacy in Multivariate Context (A) Correlation between TMB and PD-L1 expression (Spearman ρ −0.087 [95% CI −0.32 to 0.16], p = 0.48). Patients with CR/PR (n = 24) are colored in blue circles; those with SD/PD (n = 51) are colored in gray squares. (B) ROC curves for multivariate model correlation with objective response (CR/PR), with model including TMB (continuous variable), PD-L1 (continuous), histology (binary, squamous versus non-squamous), smoking status (binary, ever versus never), performance status (Eastern Cooperative Oncology Group [ECOG] 0 versus 1), and tumor burden (binary, > versus ≤ median) (plain line, AUC 0.869). Univariate correlation of TMB with objective response is shown again for reference (dotted line). (C) ROC curves for univariate correlation of TMB (continuous) with progression-free survival (dotted line) at 6 months (purple, AUC = 0.585) or 12 months (yellow, AUC = 0.558). ROC curves for multivariate correlation of model including TMB (continuous), PD-L1 (continuous), histology (squamous versus non-squamous), smoking status (ever versus never), performance status (ECOG 0 versus 1), and tumor burden (binary, > versus ≤ median) also shown (plain lines; at 6 months AUC = 0.764, at 12 months AUC = 0.831). (D) Histogram of objective response (CR/PR) to nivolumab plus ipilimumab in patients characterized by high mutation burden (>median TMB) and PD-L1 expression (≥1%), high mutation burden or PD-L1 expression, or neither. Response rates (n/N) are shown above each bar, with proportion of those with PR/CR colored in blue. Chi-square for trend p < 0.0001. See also Figure S4.

Lastly, we examined the impact of mutation burden on response in the context of tumor PD-L1 expression, which was known in 70 of 75 patients (93%). There was no correlation between PD-L1 expression and TMB (Spearman ρ −0.087, p = 0.48; Figure 3A). The distribution of TMB was similar in those with PD-L1 positive versus PD-L1 negative tumors (median 162 versus 135, Mann-Whitney p = 0.89). In multivariable analysis incorporating PD-L1 expression, histology, smoking status, performance status, and tumor burden, TMB was independently associated with ORR (p = 0.001, Figure 3B) and PFS (p = 0.002, Figures 3C and S4A). When considered in composite, patients with positive PD-L1 expression (defined as ≥1% expression) and high TMB (defined as > median) had significantly improved rates of ORR and PFS compared with those tumors with only one or neither variable (ORR chi-square for trend p < 0.0001, Figure 3D; PFS log rank for trend p = 0.0072, Figure S4B). Of particular note, four of five responders whose tumors were PD-L1 negative had high mutation burden (absolute mutation burden range 307–1175, TCGA percentile rank 72-98^th^-tile), including one with a pathologically confirmed complete response (Figure S4C).

## Discussion

In patients with advanced cancers treated with PD-1 blockade monotherapy, multiple reports across multiple tumor types (Carbone et al., 2017, Le et al., 2017, Rizvi et al., 2015, Rosenberg et al., 2016, Snyder et al., 2014, Van Allen et al., 2015) have identified an association between increased TMB and increased likelihood of disease control. However, prior to this report, the significance of TMB for predicting response to combination immunotherapy in NSCLC was not known. In fact, we initially hypothesized that TMB in patients with NSCLCs would not impact response to PD-1 plus CTLA-4 blockade therapy. In an analogous scenario, the impact of PD-L1 expression is abated in patients with melanoma treated with PD-1 plus CTLA-4 blockade (Wolchok et al., 2017), and in a report of 17 patients with melanoma treated with combination immunotherapy, TMB did not correlate with response (Goodman et al., 2017). In contrast, we found that TMB is the strongest predictor of efficacy identified in our dataset of patients with NSCLC. Similar results were seen in a recent report of patients with small cell lung cancer treated with PD-1 plus CTLA-4 blockade (Hellmann et al., 2018), suggesting the importance of TMB as biomarker for combination immunotherapy across lung cancers.

Given the correlation between TMB and efficacy, it is tantalizing to consider how TMB may be applied prospectively as a biomarker. One criticism of determining TMB by WES, as we have in this study, is that WES is currently challenging to perform in an expeditious time frame and at adequate scale needed for general use in patients with advanced NSCLC. Emerging independent sets of data have demonstrated that targeted next-generation sequencing (NGS) panels, which are already being used routinely in clinic, may provide a reasonable estimate of exonic mutation burden (Chalmers et al., 2017, Rizvi et al., 2018, Zehir et al., 2017). We found that estimated TMB using only genes covered in US Food and Drug Administration-approved targeted NGS panels MSK-IMPACT (Zehir et al., 2017) and FoundationOne (Frampton et al., 2013) were similarly predictive to the TMB derived from WES. These currently available assays may provide a practical platform for clinical practice now. Furthermore, although the technology is still being optimized, use of cell-free DNA in plasma to estimate TMB has recently been shown to be feasible and, when detectable, to correlate with TMB estimated from tumor tissue (Fabrizio et al., 2017, Gandara et al., 2017).

The mechanism(s) underlying the association between TMB and benefit with immunotherapy is not entirely clear. A leading hypothesis suggests that neoantigens, tumor-specific non-self peptides resulting from somatic nonsynonymous mutations, represent the mechanistic link. Several pre-clinical and clinical reports have described neoantigen-specific T cell responses that direct anti-tumor immunity (Gubin et al., 2014, Hadrup et al., 2009, McGranahan et al., 2016, Tran et al., 2015, van Rooij et al., 2013). Neoantigen-specific T cell responses appear to be few in numbers for any given patient, such that increased TMB may associate with increased benefit by increasing the chance for an effective neoantigen to be generated and presented. The decreased predictive strength of total, rather than nonsynonymous, TMB on efficacy found in our study supports this hypothesis by emphasizing the particular importance of nonsynonymous variants. However, the capacity to computationally predict neoantigens from exome sequences remains incomplete. In this study, similar to others, predicted neoantigen burden was largely proportional to mutation burden and did not appear to have distinct predictive power. In one study (Van Allen et al., 2015), shuffling of HLAs such that neoantigen predictions were no longer based on patient-specific HLA alleles had no impact on the association of neoantigen burden and benefit. These data suggest that current routine neoantigen prediction algorithms based on predicted peptide-HLA binding affinity alone are inadequate. Future work to incorporate more nuanced understanding of neoantigen clonality (McGranahan et al., 2016), fitness (Luksza et al., 2017), and interactions with T cell receptor binding (Glanville et al., 2017) are critical, especially as personalized vaccine strategies targeting tumor-specific predicted neoantigens are now under way in the clinic (Ott et al., 2017, Sahin et al., 2017).

Although TMB was the strongest feature associated with efficacy in this study, it remains an unrefined metric. Further refinement in understanding the molecular determinants of response to immunotherapy will likely come from identification of key somatic variants that mediate response or resistance to immunotherapy. Recent case reports of acquired resistance (Anagnostou et al., 2017, Zaretsky et al., 2016) and pre-clinical reports using CRISPR/Cas9 screening (Burr et al., 2017, Manguso et al., 2017, Patel et al., 2017) have begun to elucidate genes in both tumors and T cells that are critical to response or resistance to immunotherapy. However, mutations in each of these individual genes appear to be relatively uncommon at least in pre-treatment tissue; selective pressure from immunotherapy at acquired resistance may reveal a distinct genomic landscape. Among recurrent genes in our cohort, *TP53* was modestly associated with increased response but even more strongly associated with increased TMB. The association between *TP53* alterations and TMB was also seen in a recent report of TCGA lung adenocarcinoma data (Dong et al., 2017), and is perhaps expected given the function of *TP53* (Levine and Oren, 2009) and its known association with smoking (Pfeifer et al., 2002). *STK11* was exclusively associated with resistance, which aligns with previous reports describing the T cell excluded phenotype associated with these variants (Skoulidis et al., 2015), but did not reach statistical significance. Studies of larger size will ultimately be critical to define the landscape and frequency of “immunologic drivers” in NSCLC and other cancers.

In addition to refining the genomic features that associate with response and resistance to immune checkpoint blockade, a combination of assays may be helpful to predict outcomes with greater sensitivity and specificity. Here, similar to patients with NSCLC treated with PD-1 monotherapy, we found that efficacy was enhanced in those characterized by both high TMB and PD-L1 positivity. Considering both variables together begins to explain patients who may otherwise be considered curious exceptions (e.g., PD-L1 negative responders who have high TMB) and further improves predictive accuracy using the composite of TMB and PD-L1. The incorporation of assays, such as peripheral T cell phenotyping, T cell receptor sequencing, multiplex pathology imaging, bulk and single-cell gene expression profiling, may provide additional biological insight and clinical predictive power in the future.

We did not identify distinct molecular features of response to combination immunotherapy relative to what has been described in patients with NSCLC treated with PD-1 blockade monotherapy. It remains uncertain how PD-1 plus CTLA-4 blockade may improve response rates compared with PD-1 blockade monotherapy. Given the magnitude of benefit in patients with TMB high tumors, we speculate that response among patients with TMB high tumors is further improved with combination therapy and/or there is a modest heightening of the slope associating TMB and benefit relative to PD-1 blockade monotherapy. Important questions remain about the mechanisms of underlying benefit of combination CTLA-4 plus PD-1 blockade and how these therapies synergize in the context of high TMB. In our report, the persistently low response rate in patients who were TMB low/PD-L1 negative demonstrates that combination therapy does not overcome the barriers to response in those patients who are least likely to respond to PD-1 blockade.

Other limitations of this study include its retrospective nature, such that tissue was not available for all patients treated on this clinical trial and there was insufficient tissue to perform additional expression analysis. Importantly, however, the clinical characteristics and outcomes in the cohort analyzed here were similar to the overall group of patients treated with nivolumab plus ipilimumab on CheckMate-012.

In summary, we demonstrate that TMB strongly predicted efficacy in patients with NSCLC treated with combination PD-1 plus CTLA-4 blockade. TMB is independent of other clinicopathologic features, including PD-L1 expression. Based in part on these data, an assessment of TMB has been incorporated in a Phase III trial examining the benefit of PD-1 plus CTLA-4 blockade (CheckMate227).

## STAR★Methods

### Key Resources Table

**Table:** 

REAGENT or RESOURCE	SOURCE	IDENTIFIER
**Antibodies**		

PD-L1	Dako	Clone 28-8

**Biological Samples**		

Human tumor biopsy tissue	Memorial Sloan Kettering Cancer Center, New York, NY; H. Lee Moffit Cancer Center and Research Institute, Tampa, FL; Fox Chase Cancer Center, Philadelphia, PA; Bristol Myers Squibb, Princeton, NJ	https://clinicaltrials.gov/ct2/show/NCT01454102

**Critical Commercial Assays**		

Agilent Sure-Select Human All Exon v2.0 (44Mb) kit	Agilent	https://www.genomics.agilent.com/article.jsp?pageId=3042
Agilent Sure-Select Human All Exon v4.0 (51Mb) kit	Agilent	https://www.genomics.agilent.com/article.jsp?pageId=3042
Rapid Capture Exome (38Mb) kit	Illumina	https://support.illumina.com/sequencing/sequencing_kits/nextera-rapid-capture-exome-kit.html

**Deposited Data**		

Human sequencing data, for 43 patients with consent to share these data	This paper	https://www.ebi.ac.uk/eva/?eva-study=PRJEB24995
TCGA processed data (LUAD)	Campbell et al., 2016 (PMID 27158780)	https://portal.gdc.cancer.gov/legacy-archive/files/b2e25bdf-f2b5-4a37-b330-05251ea09f2c
TCGA processed data (LUSC)	Campbell et al., 2016 (PMID 27158780)	https://portal.gdc.cancer.gov/legacy-archive/files/d7e90ea9-49b5-4efc-9f78-bd5244cd6367

**Software and Algorithms**		

Burrows-Wheeler Aligner (BWA) version 0.5.9-tpx	Li and Durbin, 2009 (PMID 19451168)	http://maq.sourceforge.net/
Genome Analysis Toolkit (GATK) version nightly-2015-07-31-g3c929b0	DePristo et al., 2011 (PMID 21778889)	https://software.broadinstitute.org/gatk/
ContEst	Cibulskis et al., 2011 (PMID 21803805)	http://archive.broadinstitute.org/cancer/cga/contest
OxoG3	Costello et al., 2013 (PMID 23303777)	http://archive.broadinstitute.org/cancer/cga/dtoxog
MuTect version v1.1.6	Cibulskis et al., 2013 (PMID 25143287)	http://archive.broadinstitute.org/cancer/cga/mutect
Indelocator		http://archive.broadinstitute.org/cancer/cga/indelocator
Varcode v0.5.15		https://github.com/hammerlab/varcode
PyEnsembl v1.0.3		https://github.com/hammerlab/pyensembl
OptiType	Snyder et al., 2017 (PMID 28552987); Szolek et al., 2014 (PMID 25143287)	https://github.com/FRED-2/Optitype
Topiary	Nathanson et al., 2017 (PMID 27956380)	https://github.com/hammerlab/topiary/
ABSOLUTE	Carter et al., 2012 (PMID 22544022)	http://archive.broadinstitute.org/cancer/cga/absolute
NetMHCcons	Karosiene et al., 2012 (PMID 22009319)	http://www.cbs.dtu.dk/services/NetMHCcons/
MutSigCV	Lawrence et al., 2013 (PMID 23770567);	http://software.broadinstitute.org/cancer/software/genepattern/modules/docs/MutSigCV
GraphPad Prism v.6	GraphPad Software	https://www.graphpad.com/
R 3.3.2	R software	https://www.r-project.org/

### Contact for Reagent and Resource Sharing

Further information and requests for resources should be directed to and will be fulfilled by the Lead Contact, Matthew Hellmann (hellmanm@mskcc.org).

### Experimental Model and Subject Details

#### Combination Immunotherapy Treated Patients

All patients had stage IV non-small cell lung cancer (NSCLC) and were treated on CheckMate 012 (NCT01454102 (Hellmann et al., 2017)) (Table S1 and Table S2). All patients initiated therapy between February 2013 and March 2015 and were treated with a combination of nivolumab and ipilimumab. All patients consented to an Institutional Review Board-approved study protocol for treatment, tissue collection, and biomarker analysis at institutions that participated in CheckMate 012 (Memorial Sloan Kettering Cancer Center, H Lee Moffitt Cancer Center, Fox Chase Cancer Center, UCLA, Jonsson Comprehensive Cancer Center, Jonsson Comprehensive Cancer Center, Yale Comprehensive Cancer Center, Sidney Kimmel Comprehensive Cancer Center at Johns Hopkins, Duke University Medical Center, UT Southwestern Medical Center, University of Washington, Juravinski Cancer Centre, McMaster University, Princess Margaret Cancer Centre, University of Toronto, Ottawa Hospital Cancer Centre, University of Ottawa). PD-L1 expression was assessed by immunohistochemistry using a previously validated rabbit anti-human anti-PD-L1 monoclonal antibody (clone 28-8; Epitomics, Berlingame, CA, USA). Quantification of tumor membranous PD-L1 expression was performed centrally on pre-treatment tumor tissue submitted as part of the clinical trial using an analytically validated automated assay developed by Dako (Carpinteria, CA, USA). A minimum of 100 evaluable tumor cells were required for determination of PD-L1 expression. PD-L1 scoring was available in 70 of 75 patients; five had unknown expression.

#### Clinical Efficacy Analyses

Per protocol, tumor assessments were collected at week 10, week 17, week 23, and then every 12 weeks until progression. Objective response was assessed by investigator-assessed RECIST v1.1 (Eisenhauer et al., 2009). Partial and complete responses were confirmed by repeat imaging occurring at least 4 weeks after the initial identification of response; unconfirmed partial responses were considered stable disease. Patients with confirmed complete or partial response were considered responders; patients with stable disease, progressive disease, or not evaluable were considered non-responders.

We also used a related outcome metric, durable clinical benefit (DCB), which we have previously described (Rizvi et al., 2015, Rizvi et al., 2018). DCB was defined as stable disease or partial response lasting longer than 6 months; all other patients were considered to have no durable benefit (NDB).

Progression-free survival was assessed as previously described (Hellmann et al., 2017), with outcomes determined as of the September 2016 database lock.

#### Tumor and Germline Samples

All tumor tissue used for sequencing was obtained prior to dosing with combination immunotherapy, with the exception of one non-responder whose tissue was collected 122 days after beginning therapy (ID# 4). The presence of tumor tissue in the sequenced samples was confirmed by examination of a representative hematoxylin and eosin-stained slide by thoracic pathologist (N.R.) or central pathology vendor through Mosaic Labs. Germline DNA was obtained from peripheral blood mononuclear cells from all patients.

### Method Details

#### Whole Exome Capture and Sequencing

Whole exome capture libraries were constructed using the Agilent Sure-Select Human All Exon v2.0 (44Mb), v4.0 (51Mb), or Illumina’s Rapid Capture Exome (38Mb) baited target kit. Enriched exome libraries were sequenced on a HiSeq 2000, 2500, or 4000 platform (Illumina, San Diego, California) to generate paired-end reads (2x76bp) to a goal of 150X mean target coverage (n=70 sequenced at the Broad Institute, Cambridge, MA; n=5 sequenced at Memorial Sloan Kettering Cancer Center Genomic Core, New York, NY).

#### Exome Alignment and Assembly

For each case, a BAM file was produced by aligning tumor and normal sequences to the hg19 human genome build using the Burrows-Wheeler Aligner (BWA) version 0.5.9-tpx (Li and Durbin, 2009). Further indel realignment, base-quality score recalibration, and duplicate-read removal were performed using the Genome Analysis Toolkit (GATK) version nightly-2015-07-31-g3c929b0 (DePristo et al., 2011).

#### Sequencing Quality Control

Quality control metrics were computed using the Broad Institute Picard software. Fingerprint genotypes were used to verify match of tumor and normal samples. Potential contamination was estimated using ContEst (Cibulskis et al., 2011). Artifacts produced by oxidation during DNA sequencing were removed using the OxoG3 filter (Costello et al., 2013). Samples with mean target coverage <60X in tumor or <30X in normal were excluded (Table S3).

#### Variant Calling

MuTect version v1.1.6 (Cibulskis et al., 2013) was used to generate single nucleotide variant (SNV) calls using default parameters. Indelocator (http://archive.broadinstitute.org/cancer/cga/indelocator) was used to generate indel calls. Mutations with variant allelic fraction < 0.05 in tumor were excluded (Taylor-Weiner et al., 2016). Site based artifact filtering was applied to mutations with variant alleles that were present in an independent panel of normal exomes derived from blood samples of non-cancer patients. Variants were annotated by Varcode (v. 0.5.15, https://github.com/hammerlab/varcode) and PyEnsembl (v. 1.0.3, https://github.com/hammerlab/pyensembl) using Ensembl Release 75.

#### Mutation Burden Quantification

Tumor mutation burden (TMB) was defined as the number of nonsynonymous alterations (SNVs or indels) for each patient (Table S3).

#### Mutation Burden Percentile Rank Compared to NSCLC Tumors Sequenced by TCGA

To compare the overall spectrum of TMB and determine the percentile rank of TMB of patients sequenced in this study relative to the TMB seen in larger series of NSCLCs, MAF files of called variants were retrieved from tumors analyzed as part of the lung adenocarcinoma and lung squamous cell carcinoma by The Cancer Genome Atlas (TCGA) projects (Campbell et al., 2016). Mutations with VAF < 0.05 were excluded. For TCGA participants with multiple samples, a single sample was chosen. To reflect a typical histologic distribution of NSCLC, we used all LUAD participants present in the MAF files (n=569) and selected a subset of available LUSC participants (n=141 (29%) of 491 participants) to create a set of 710 samples comprised of 80% adenocarcinoma and 20% squamous cell carcinoma (Table S3). The TMB of samples in this study were then compared to the TMB of the NSCLC TCGA cohort to assign each sample a percentile rank.

#### HLA Analysis

Four-digit class I HLA alleles were inferred using OptiType (https://github.com/FRED-2/Optitype) (Snyder et al., 2017, Szolek et al., 2014).

#### In Silico Neoantigen Prediction Pipeline

Using Topiary (https://github.com/hammerlab/topiary/) (Nathanson et al., 2017), the mutated DNA sequences were virtually translated into corresponding mutated peptide sequences. Topiary was used to run NetMHCcons (v. 1.1) (Karosiene et al., 2012) in order to predict MHC class I binding affinity for all 8 to 11mer peptide sequences containing the mutated amino acid. For variants longer than a single residue, all 8-11mers downstream of the variant were considered. Candidate neoantigens were those peptides with binding affinity IC50 of ≤500nM to one (or more) of the patient-specific HLA alleles (Table S3); strong binding candidate neoantigens were considered those with binding affinity IC50 of ≤50nM.

#### Predicted Neoantigen Clonality

To determine the cancer cell fraction (CCF) of each mutation, we integrated the variant allele frequency with the local copy number with purity and ploidy estimates. Each alteration, the variant allele frequency (VAF) depends on the local copy number of the tumor (CPNmut), the purity (p), the local copy number of the normal sample (CPNnorm) and also the cancer cell fraction (CCF), defined as the proportion of cancer cells harboring the mutations. The expected VAF, given the CCF, can be calculated as follows: CAF (CCF) = p^∗^CCF / CPNnorm (1-p) + p^∗^CPNmut.

For a given mutation with ‘a’ alternative reads, and a depth of ‘N’, the probability of a given CCF can be estimated using a binomial distribution P(CCF) = binom(aIN, VAF(CCF)). CCF values can then be calculated over a uniform grid of 100 CCF values (0.01, 1) and subsequently normalized to obtain a posterior distribution. To avoid overestimating the number of clonal alterations, we classified mutations as clonal if there was >0.5 probability that the cancer cell fraction was >0.95 (Landau et al., 2013). Clonality could be resolved in all but two patients. We restricted clonality estimation to single nucleotide variants, with copy number as assessed by ABSOLUTE (Carter et al., 2012).

#### Recurrently Altered Genes

MutSigCV (version 1.4,(Lawrence et al., 2013)) was used to identify recurrently altered genes in both the IpiNivo cohort as well as the NSCLC TCGA cohort (Table S4). Genes with q values <0.1 were considered to be significantly recurrently mutated.

### Quantification and Statistical Analyses

Differences in TMB between two groups were examined using the non-parametric Mann-Whitney test. Fisher’s exact test was used to compare proportions between two groups, or chi-square test for three groups. For progression-free survival analysis, the log-rank test was used to compare Kaplan-Meier survival curves and the Mantel-Haenszel method was used to determine hazard ratios between groups. Correlations were examined by the Spearman correlation method. Receiver operator characteristic (ROC) curves plotting sensitivity and 1-specificity of continuous variables were assessed by generating the area under the curve; p-values were also reported. An analysis of enrichment in frequency of altered genes of *a priori* significance (Table S4) were examined using Odds ratio and Fisher’s exact test. The frequency of recurrently altered genes identified by MutSigCV were compared in responder vs non-responder groups as well as high TMB vs low TMB groups using odds ratio and Fisher’s exact text. All reported p-values are two sided. Correlations between mutation burden and PD-L1 expression were determined using the Spearman correlation formula. Multivariable logistic and Cox regression were conducted to assess the impact of TMB on ORR and PFS, respectively, while adjusting for other covariates described. Statistical analyses were performed using GraphPad Prism v.6 and R 3.3.2.

### Data and Software Availability

Our dataset, for the 43 patients with consent to share these sequencing data, is deposited in the European Variation Archive. The accession number for the sequencing data is PRJEB24995, https://www.ebi.ac.uk/eva/?eva-study=PRJEB24995.

## Supplemental Information

Document S1. Figures S1–S4 and Table S1

Table S2. Clinical Characteristics, Outcomes, and Summary Molecular Features of Individual Patients, Related to Table 1

Table S3. Genomic Characteristics, Related to Figures 1 and 2

Table S4. Associations of Individual Altered Genes with Outcomes and TMB, Related to Figure 2

Document S2. Article plus Supplemental Information
