# Supplementary material for: Genomic Features of Response to Combination Immunotherapy in Patients with Advanced Non-Small-Cell Lung Cancer
Source: Cancer Cell. 2018 May 14;33(5):843–852.e4. doi: 10.1016/j.ccell.2018.03.018 (PMC5953836; doi:10.1016/j.ccell.2018.03.018)
Supplement: Document S1. Figures S1–S4 and Table S1 [file mmc1.pdf]

Cancer Cell, Volume *33*

Supplemental Information

Genomic Features of Response to Combination Immunotherapy in Patients with Advanced Non-Small-Cell Lung Cancer

Matthew D. Hellmann, Tavi Nathanson, Hira Rizvi, Benjamin C. Creelan, Francisco Sanchez-Vega, Arun Ahuja, Ai Ni, Jacki B. Novik, Levi M.B. Mangarin, Mohsen Abu-Akeel, Cailian Liu, Jennifer L. Sauter, Natasha Rekhtman, Eliza Chang, Margaret K. Callahan, Jamie E. Chaft, Martin H. Voss, Megan Tenet, Xue-Mei Li, Kelly Covello, Andrea Renninger, Patrik Vitazka, William J. Geese, Hossein Borghaei, Charles M. Rudin, Scott J. Antonia, Charles Swanton, Jeff Hammerbacher, Taha Merghoub, Nicholas McGranahan, Alexandra Snyder, and Jedd D. Wolchok

Table S1, related to Table 1: Clinical characteristics of all patients in CheckMate-012 by whether or not profiled by whole exome sequencing.

	Overall Cohort (n=197)		WES cohort (n=75)		Non-WES cohort (n=122)		
**Patient Characteristics**	**No.**	**(%)**	**No.**	**(%)**	**No.**	**(%)**	**p value***
Median age, years (range)	63	(37-91)	66	(42-87)	63	(37-91)	0.5864
Gender							0.7692
Male	101	(51)	37	(49)	64	(52)	
Female	96	(49)	38	(51)	58	(48)	
Histology							0.8612
Non-squamous	153	(78)	59	(79)	94	(77)	
Squamous	44	(22)	16	(21)	28	(23)	
Smoking Status^							0.3060
Current/Former	148	(76)	60	(80)	88	(73)	
Never	48	(24)	15	(20)	33	(27)	
Performance Status							0.4450
ECOG 0	71	(36)	30	(40)	41	(34)	
ECOG 1	126	(64)	45	(60)	81	(66)	
PD-L1 expression							0.8699#
0%	56	(28)	25	(33)	33	(27)	
≥ 1%	108	(55)	45	(60)	64	(52)	
Unknown	33	(17)	5	(7)	25	(21)	
Best Overall Response							0.5597
Complete/Partial Response	63	(32)	24	(32)	39	(32)	
Stable Disease	63	(32)	27	(36)	36	(29)	
Progression/Not evaluable	71	(36)	24	(32)	47	(39)	
Clinical Benefit							0.3013
Durable Clinical Benefit (DCB)	87	(44)	37	(49)	50	(41)	
No durable benefit (NDB)	110	(56)	38	(51)	72	(59)	

*Comparison of WES cohort vs Non-WES cohort using Fisher's exact test, except age (t test) and BOR (Chi-square test)
^Smoking status was not reported for one patient in the Non-WES cohort
#Reflects comparison of PD-L1 0% vs ≥ 1%

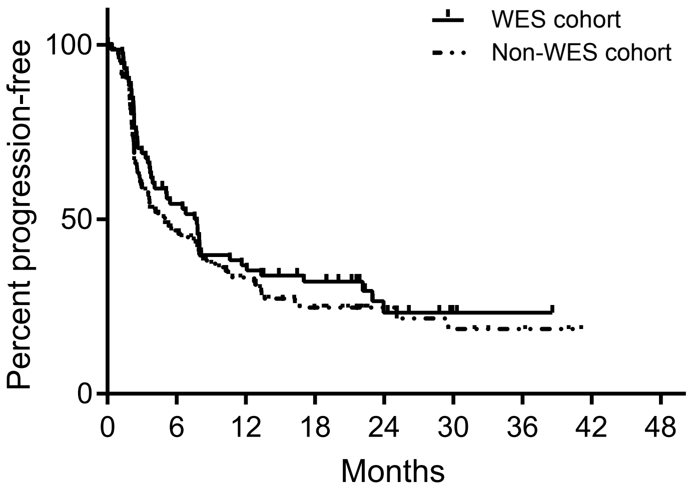

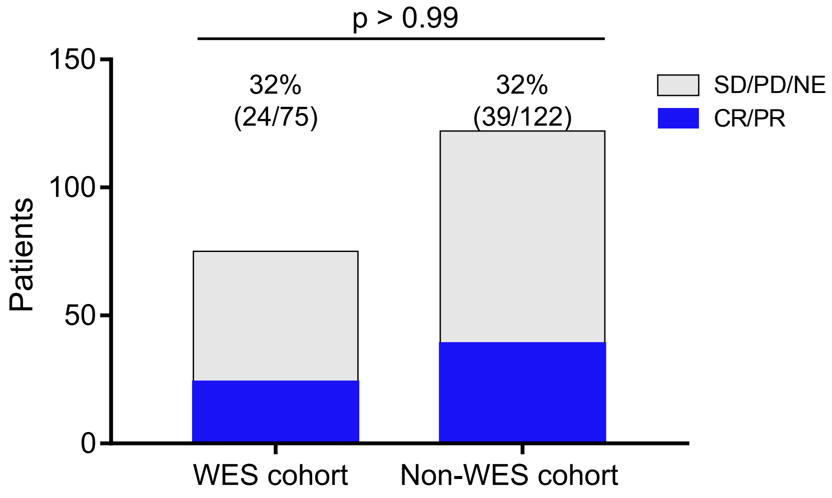

Figure S1, related to Table 1: Progression-free survival (PFS, top) and objective response rate (ORR, bottom) of the WES cohort (n = 75) vs other patients treated (non-WES, n = 122) with nivolumab plus ipilimumab as part of CheckMate-012. PFS was similar among the WES cohort and non-WES cohort (log-rank p = 0.30) (top panel). The proportion of responders was similar in the WES cohort vs non-WES cohort (Fisher's exact p > 0.99) (bottom panel). Percentages and rate (n/N) of responders in each group are reported above each bar and colored in blue.

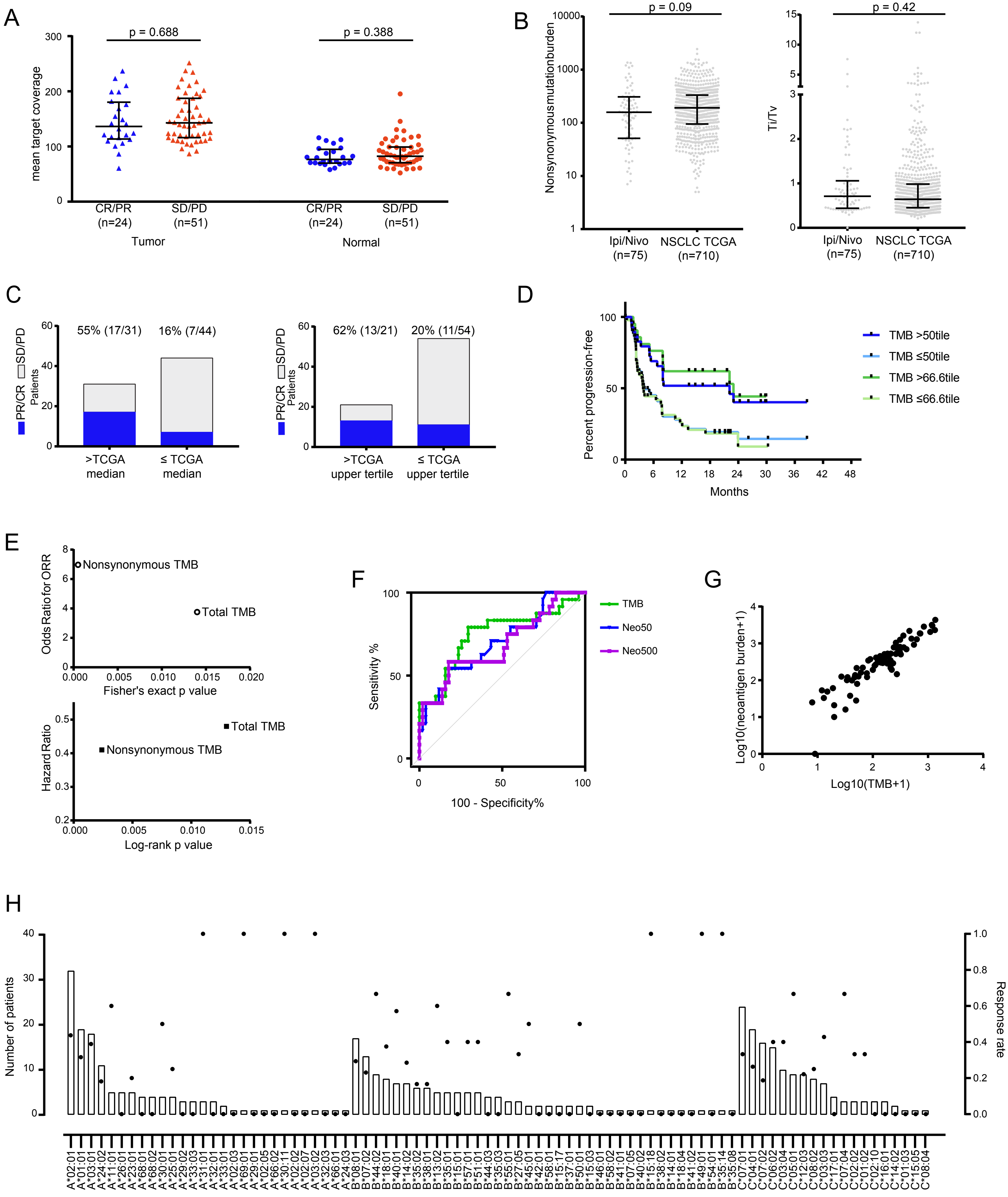
A
p = 0.688
p = 0.388
mean target coverage
300
200
100
0
CR/PR (n=24)
SD/PD (n=51)
Tumor
CR/PR (n=24)
SD/PD (n=51)
Normal
B
p = 0.09
Nonsynonymous mutation burden
10000
1000
100
10
1
Ipi/Nivo (n=75)
NSCLC TCGA (n=710)
p = 0.42
Ti/Tv
15
10
5
3
2
1
0
Ipi/Nivo (n=75)
NSCLC TCGA (n=710)
C
PR/CR SD/PD Patients
60
40
20
0
55% (17/31)
16% (7/44)
>TCGA median
≤ TCGA median
62% (13/21)
20% (11/54)
>TCGA upper tertile
≤ TCGA upper tertile
D
Percent progression-free
100
50
0
0 6 12 18 24 30 36 42 48
Months
TMB >50tile
TMB ≤50tile
TMB >66.6tile
TMB ≤66.6tile
E
Odds Ratio for ORR
Nonsynonymous TMB
Total TMB
0.000 0.005 0.010 0.015 0.020
Fisher's exact p value
Hazard Ratio
Total TMB
Nonsynonymous TMB
0.000 0.005 0.010 0.015
Log-rank p value
F
Sensitivity %
100
50
0
0 50 100
100 - Specificity%
TMB
Neo50
Neo500
G
Log10(neoantigen burden+1)
Log10(TMB+1)
0 1 2 3 4
H
Number of patients
40
30
20
10
0
Response rate
1.0
0.8
0.6
0.4
0.2
0.0
A*02:01 A*01:01 A*03:01 A*24:02 A*11:01 A*26:01 A*23:01 A*68:01 A*68:02 A*30:01 A*25:01 A*29:02 A*33:03 A*31:01 A*32:01 A*33:01 A*02:03 A*69:01 A*29:01 A*02:05 A*66:02 A*30:11 A*02:07 A*03:02 A*32:03 A*66:01 A*24:03
B*08:01 B*07:02 B*44:02 B*18:01 B*40:01 B*14:02 B*35:02 B*38:01 B*13:02 B*35:01 B*15:01 B*57:01 B*51:01 B*44:03 B*35:03 B*55:01 B*27:05 B*45:01 B*42:01 B*58:01 B*15:17 B*37:01 B*50:01 B*15:03 B*46:01 B*58:02 B*41:01 B*07:05 B*40:02 B*15:18 B*38:02 B*14:01 B*18:04 B*41:02 B*49:01 B*54:01 B*35:14 B*35:08
C*07:01 C*04:01 C*07:02 C*06:02 C*03:04 C*05:01 C*12:03 C*08:02 C*03:03 C*17:01 C*07:04 C*02:02 C*01:02 C*02:10 C*16:01 C*14:02 C*01:03 C*15:05 C*08:04

Figure S2, related to Figure 1 and Figure 2: (A) Mean target coverage (MTC) of tumor and matched normal by response category. MTC among responders (CR/PR) was similar to non-responders (SD/PD) for both tumor (136.3X vs 142.9X, p = 0.688) and normal (76.64X vs 82.34X, p = 0.388). Median and interquartile range are shown by the black bars. **(B)** The range of nonsynonymous mutation burden among NSCLCs profiled by WES in this study and NSCLCs profiled by TCGA project are shown in the left panel. Mutation burden was similar in this cohort (median 158 mutations [interquartile range (IQR) 51-307]) compared to the TCGA cohort (median 191 mutations [IQR 95-332], p = 0.09). The right panel shows the distribution of transition/transversion ratio (Ti/Tv) in each group. The median transition/ transversion (Ti/Tv) ratio among this cohort (0.71 [IQR 0.44-1.06]) was comparable to the TCGA cohort (0.64 [IQR 0.45-0.99], p = 0.42). Black bars represent the median and interquartile range. **(C)** Objective response (CR/PR, blue) of the upper half or upper tertile of TMB among NSCLCs profiled by TCGA. The median TMB in NSCLC was 191 mutations and upper tertile was 266 mutations. Objective response was significantly greater among patients above the median (55% vs 16%, Odds ratio = 6.42 [95% CI 2.15-17.56], Fisher's exact p = 0.0008) and upper tertile (62% vs 21%, Odds ratio = 6.35 [95% CI 2.24-17.56], Fisher's exact p = 0.0009) compared to below, respectively. Percentages and rates (n/N) are shown above each bar. **(D)** PFS for patients with TMB above vs below the median and upper tertile was significantly improved (stratified by 50th percentile, median PFS 22.1 mo vs 3.8 mo, Mantel-Haenszel HR 0.44, log-rank p = 0.0047; stratified by 66th percentile, median PFS 23.0 mo vs 4.1 mo, Mantel-Haenszel HR 0.42, log-rank p = 0.0028). **(E)** Impact of nonsynonymous TMB versus total TMB (inclusive of silent variants) on outcomes. The Odds ratio for response above versus below the respective median TMBs was higher and more significant when using nonsynonymous variants relative to all variants (nonsynonymous TMB Odds ratio 6.97 [95% CI 2.19-19.02], Fisher's exact p = 0.0005; total TMB Odds ratio 3.76 [95% CI 1.33-9.76], Fisher's exact p = 0.014). The hazard ratio for PFS above versus below the respective median TMBs was higher and more significant when using nonsynonymous TMB relative to total TMB (nonsynonymous TMB Mantel-Haenszel HR 0.41, Log-rank p = 0.0024; total TMB Mantel-Haenszel HR 0.48, Log-rank p = 0.013). **(F)** ROC curves of objective response rate (CR/PR) using TMB (green line) (AUC = 0.75 [95% CI 0.62-0.88], p = 0.006), neoantigen MHC binding affinity <50 nM (blue line) (AUC = 0.70 [95% CI 0.57-0.83], p = 0.0051), and neoantigen MHC binding affinity <500 nM (purple line) (AUC = 0.69 [95% CI 0.55-0.82], p = 0.0093). **(G)** Scatter plot depicting correlation of tumor mutation burden and neoantigen burden (spearman rho = 0.92 [95% CI 0.88-0.95], p < 0.0001). Each dot represents an individual patient. Both variables are shown as log10(X+1) transformations, with +1 added to incorporate one patient with zero predicted neoantigens. **(H)** Association with HLA alleles and efficacy. The histogram depicts the number of patients with each HLA allele, grouped as HLA-A, B, and C, quantified by the left axis. The dot above each bar reflects the objective response among patients with each HLA allele, corresponding to the right axis. No clear pattern of association between HLA allele and response rate is evident.

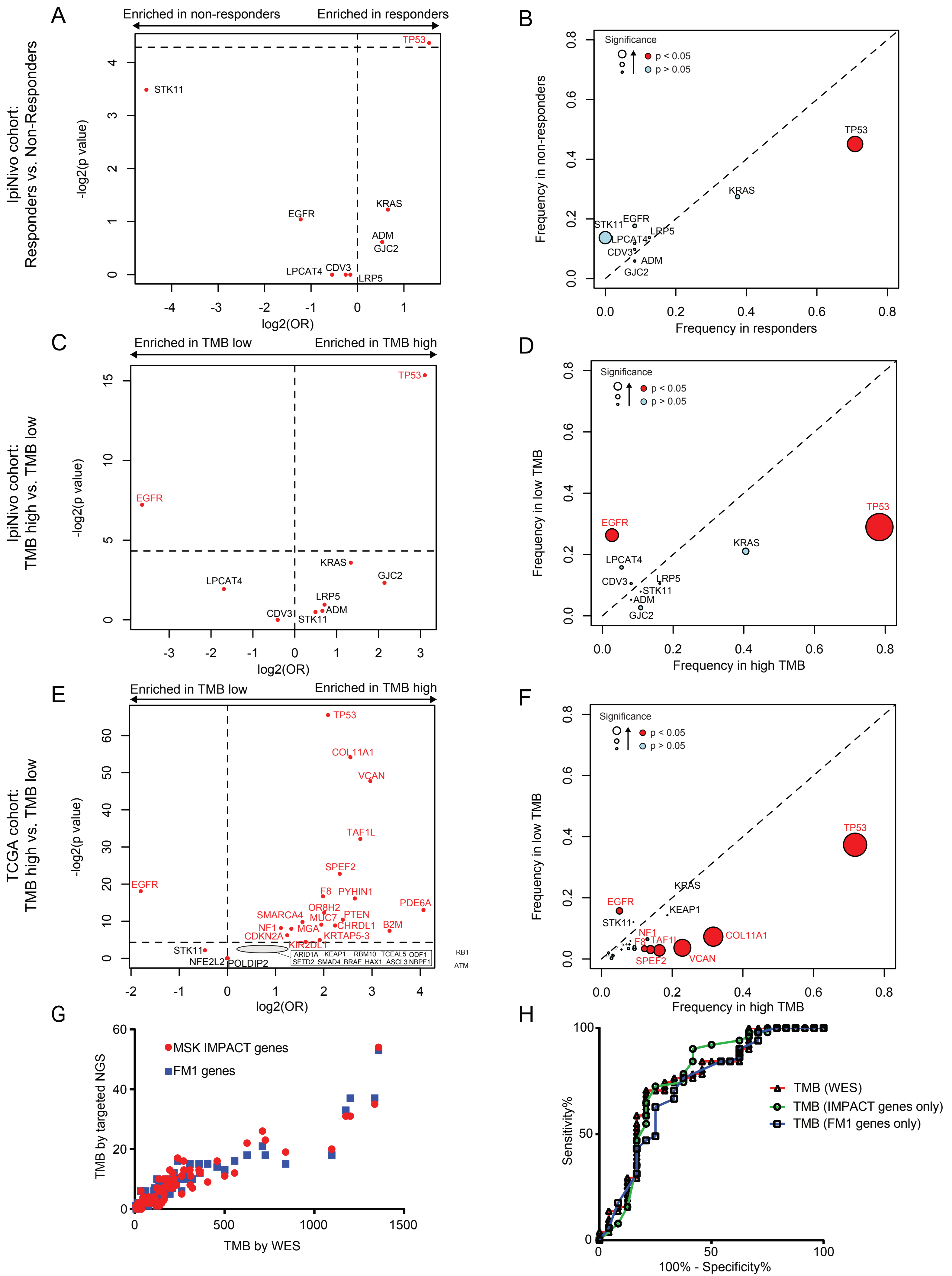
A
IpiNivo cohort:
Responders vs. Non-Responders
Enriched in non-responders
Enriched in responders
-log2(p value)
log2(OR)
TP53
STK11
EGFR
KRAS
ADM
GJC2
LPCAT4
CDV3
LRP5
B
Significance
p < 0.05
p > 0.05
Frequency in non-responders
Frequency in responders
TP53
KRAS
STK11
EGFR
LRP5
LPCAT4
CDV3
ADM
GJC2
C
IpiNivo cohort:
TMB high vs. TMB low
Enriched in TMB low
Enriched in TMB high
-log2(p value)
log2(OR)
TP53
EGFR
KRAS
GJC2
LPCAT4
LRP5
ADM
CDV3
STK11
D
Significance
p < 0.05
p > 0.05
Frequency in low TMB
Frequency in high TMB
TP53
EGFR
KRAS
LPCAT4
CDV3
LRP5
STK11
ADM
GJC2
E
TCGA cohort:
TMB high vs. TMB low
Enriched in TMB low
Enriched in TMB high
-log2(p value)
log2(OR)
TP53
COL11A1
VCAN
TAF1L
SPEF2
EGFR
F8
PYHIN1
PDE6A
OR8H2
SMARCA4
MUC7
PTEN
NF1
MGA
CHRDL1
B2M
CDKN2A
KRTAP5-3
KIR2DL1
STK11
NFE2L2
POLDIP2
ARID1A KEAP1 RBM10 TCEAL5 ODF1
SETD2 SMAD4 BRAF HAX1 ASCL3 NBPF1
RB1
ATM
F
Significance
p < 0.05
p > 0.05
Frequency in low TMB
Frequency in high TMB
TP53
KRAS
EGFR
KEAP1
STK11
NF1
F8
TAF1L
COL11A1
VCAN
SPEF2
G
MSK IMPACT genes
FM1 genes
TMB by targeted NGS
TMB by WES
H
TMB (WES)
TMB (IMPACT genes only)
TMB (FM1 genes only)
Sensitivity%
100% - Specificity%

Figure S3, related to Figure 2: Analysis of recurrently altered genes identified by MutSigCV in ipi/nivo and NSCLC TCGA cohorts**. (A)** Plot showing log odd ratios (x axis) vs. log p values (y axis) for a comparison of frequency of recurrently altered genes in responders vs. non-responders (p value using Fisher's exact test). **(B)** Plot showing frequency of occurrence in responders (x axis) vs. non-responders (y axis). Only genes that had been found as recurrently mutated at statistically significant levels based on MutSigCV analysis are included in the analysis. Genes with $p<0.05$ are colored in red. **(C,D)** Same as **(A,B)** but comparing high TMB vs. low TMB samples (> vs ≤median of ipi/nivo cohort). **(E,F)** Same as **(C,D)**, but using recurrently altered genes identified in the NSCLC TCGA cohort and comparing high TMB vs. low TMB samples (> vs ≤median of NSCLC TCGA cohort). **(G)** TMB quantified by WES correlates with the estimate of TMB using *in silico* filtering of genes represented in the MSK-IMPACT or FoundationOne panels (MSK-IMPACT Spearman rho 0.91 [95% CI 0.85-0.94], $p < 0.0001$; FM1 Spearman rho 0.93 [95% CI 0.88-0.95], $p < 0.0001$). Each dot represents an individual patient. **(H)** ROC curves depicting the correlation of objective response with TMB by WES (AUC = 0.75 [95% CI 0.62-0.88], p = 0.0006) and TMB estimated using genes represented in the MSK-IMPACT panel (AUC = 0.76 [95% CI 0.62-0.89], p = 0.0003) or FoundationOne Panel (AUC = 0.72 [95% CI 0.59-0.85], p = 0.002).

A

ORR

Model	Covariates	Odds Ratio	p value	AUC
Univariate	Mutation burden (binary, > vs ≤ median)	6.97	**<0.001**	0.719
Multivariate	Mutation burden (binary, > vs ≤ median)	7.86	**0.003**	0.838
	PD-L1 (> 0 vs 0)	3.37	0.075	
	Histology (SQ vs non-SQ)	1.50	0.611	
	Smoking (ever vs never)	4.16	0.271	
	Performance status (ECOG 0 vs 1)	3.24	0.073	
	Tumor burden (binary, > vs ≤ median)	0.45	0.230	

Model	Covariates	Odds Ratio	p value	AUC
Univariate	Mutation burden (continuous)	1.004	**0.002**	0.748
Multivariate	Mutation burden (continuous)	1.005	**0.001**	0.869
	PD-L1 (continuous)	1.03	**0.024**	
	Histology (SQ vs non-SQ)	1.58	0.592	
	Smoking (ever vs never)	22.6	0.139	
	Performance status (ECOG 0 vs 1)	1.82	0.409	
	Tumor burden (continuous)	0.99	0.283	

PFS

Model	Covariates	Odds Ratio	p value	AUC
Univariate	Mutation burden (binary, > vs ≤ median)	0.42	**0.003**	0.61
Multivariate	Mutation burden (binary, > vs ≤ median)	0.37	**0.004**	0.659
	PD-L1 (> 0 vs 0)	1.05	0.892	
	Histology (SQ vs non-SQ)	0.82	0.620	
	Smoking (ever vs never)	0.87	0.722	
	Performance status (ECOG 0 vs 1)	0.65	0.199	
	Tumor burden (binary, > vs ≤ median)	1.44	0.252	

Model	Covariates	Odds Ratio	p value	AUC
Univariate	Mutation burden (continuous)	0.998	**0.004**	0.61
Multivariate	Mutation burden (continuous)	0.998	**0.002**	0.711
	PD-L1 (continuous)	0.99	**0.030**	
	Histology (SQ vs non-SQ)	0.77	0.531	
	Smoking (ever vs never)	0.74	0.448	
	Performance status (ECOG 0 vs 1)	0.71	0.323	
	Tumor burden (continuous)	1.01	0.067	

B

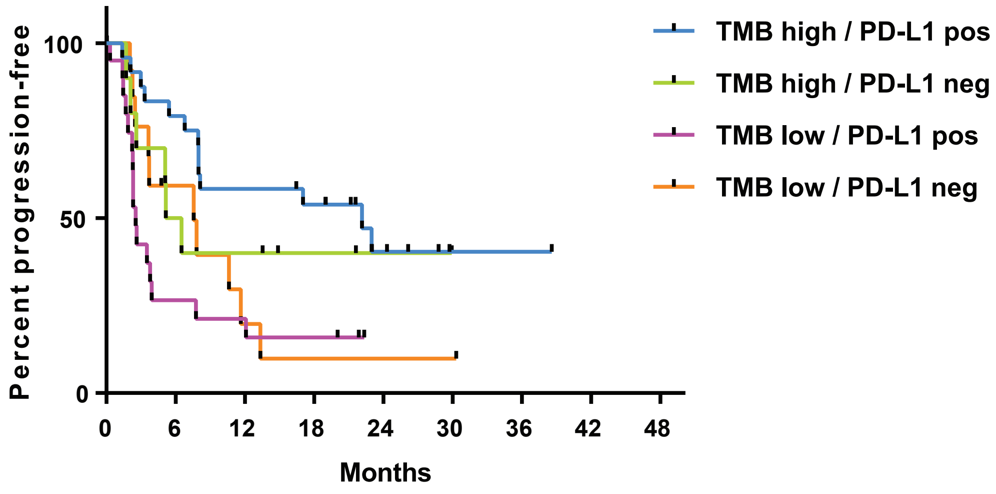

C

Before Nivolumab plus Ipilimumab Therapy

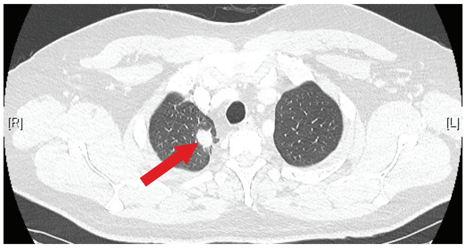

Following Nivolumab plus Ipilimumab Therapy

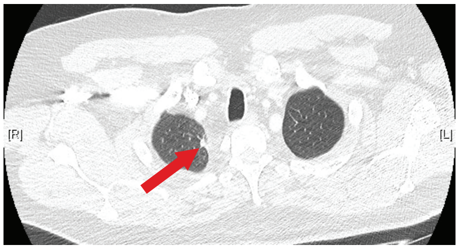

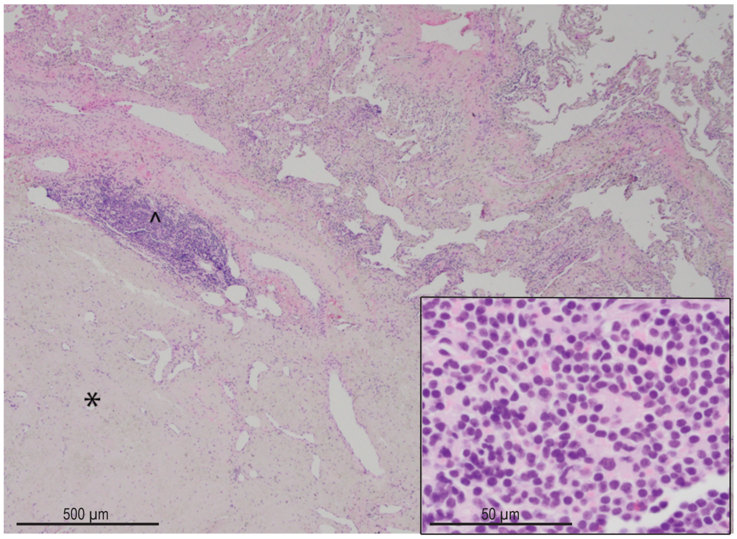

Figure S4, related to Figure 3: **(A)** Tables for multivariate analysis of TMB on ORR and PFS using mutation burden, PD-L1 expression, histology, smoking status, performance status, and tumor burden (sum unidirectional size of target lesions). In a univariate analysis using mutation burden (as a binary or continuous variable), both objective response and progression-free survival significantly associate with TMB. In a multivariate analysis, significant associations remained with respect to TMB for both ORR and PFS. **(B)** Progression-free survival of patients with TMB and PD-L1 expression as a composite variable (log-rank for trend $p = 0.0057$). TMB high is defined as > median and PD-L1 positive is defined as ≥ 1% tumor expression. **(C)** Case of patient whose tumor is PD-L1 negative (0% tumor expression) and TMB high (840 nonsynonymous mutations, 95th percentile of TCGA NSCLCs) who achieved a complete pathologic response to nivolumab plus ipilimumab. Top panel shows lesion prior to and following initiation of therapy. The bottom panel shows representative histology at time of resection of remaining residual disease. The tumor bed (40x; H&E stain) is characterized by fibroelastotic scar (*) with adjacent aggregates of chronic inflammatory cells at the periphery (^). The aggregates of inflammatory cells are composed predominantly of lymphocytes (inset; 400x).
